# Diplopia and Exophthalmos As Clinical Manifestations of Sinonasal Undifferentiated Carcinoma: A Case Report

**DOI:** 10.7759/cureus.109816

**Published:** 2026-05-28

**Authors:** Emily Ha, Ashlyn S Everett

**Affiliations:** 1 Radiation Oncology, Alabama College of Osteopathic Medicine, Dothan, USA; 2 Radiation Oncology, Alliance Cancer Care, Huntsville, USA

**Keywords:** epithelial cancer, ethmoid sinus mass, hematology-oncology, induction chemotherapy, intracranial extension, oncology, radiation oncology, sinonasal malignancy, sinonasal undifferentiated carcinoma, snuc

## Abstract

Sinonasal undifferentiated carcinoma (SNUC) is a rare and highly aggressive epithelial malignancy with an incidence of 0.2 per 100,000 individuals, often presenting at advanced stages due to nonspecific sinonasal symptoms. Orbital involvement may serve as an important early clinical clue in its diagnosis.

A 72-year-old man presented with progressive unilateral exophthalmos, excessive lacrimation, and intermittent diplopia. Imaging revealed an extensive sinonasal mass with orbital involvement. Histopathologic evaluation confirmed the diagnosis of SNUC of the ethmoid sinus, classified according to the American Joint Committee on Cancer 8th edition staging system for sinonasal malignancies as cT4N0M0 (Stage IVA). Due to extensive local invasion with orbital involvement, multidisciplinary consensus favored induction chemotherapy followed by definitive concurrent chemoradiation rather than surgical resection in an effort to preserve orbital function and avoid significant operative morbidity. The patient was treated with induction chemotherapy followed by definitive concurrent chemoradiation, resulting in a complete radiographic and metabolic response. At long-term follow-up, he remains disease-free with manageable treatment-related toxicities.

This case highlights the importance of recognizing orbital symptoms as early manifestations of SNUC. It supports the role of induction chemotherapy as part of a multimodal treatment strategy for locally advanced disease.

## Introduction

Sinonasal undifferentiated carcinoma (SNUC) is a rare and highly aggressive malignancy arising from the Schneiderian epithelium of the sinonasal tract. First described by Frierson et al. in 1986, SNUC accounts for approximately 3-5% of sinonasal cancers and has an estimated incidence of approximately 0.2 cases per 100,000 individuals annually [1-3]. Despite its rarity, SNUC is clinically significant due to its aggressive biologic behavior, rapid progression, and historically poor prognosis. The tumor is characterized by extensive local invasion, a high proliferative index, and a strong propensity for early invasion into adjacent structures, including the orbit, skull base, and intracranial compartment [4].

Patients with SNUC commonly present with nonspecific sinonasal symptoms, including nasal obstruction, epistaxis, facial pressure, or headache. These symptoms frequently mimic benign inflammatory conditions, causing delays in diagnosis. Subsequently, the majority of patients present with locally advanced disease at the time of detection [2-3]. Advanced-stage tumors may demonstrate invasion of surrounding structures, including the orbit, anterior cranial fossa, and pterygopalatine fossa, contributing to significant morbidity and complicating treatment strategies [3].

Orbital manifestations, including exophthalmos, diplopia, or visual disturbances, are relatively uncommon initial complaints but may represent important indicators of tumor extension beyond the sinonasal cavity. When present, these symptoms often reflect invasion of adjacent structures and may signal advanced disease that requires complex, multidisciplinary management [4]. 

Recognition of such atypical presentations is critical, as early diagnostic evaluation and timely initiation of therapy may improve outcomes and allow for organ-preserving treatment approaches. Morphological overlap with other sinonasal malignancies, including olfactory neuroblastoma, nasopharyngeal carcinoma, and sinonasal neuroendocrine tumors, contributes to the challenge of diagnosis. Therefore, immunohistochemical analysis plays a key role in distinguishing SNUC from these entities and confirming the diagnosis [5].

Due to the absence of standardized treatment guidelines and the aggressive nature of the disease, management of SNUC continues to be a challenge. Current management involves multimodal therapy incorporating combinations of surgery, chemotherapy, and radiation therapy. More recently, induction chemotherapy has emerged as an important component of treatment for locally advanced disease, allowing clinicians to assess tumor chemosensitivity, reduce tumor burden, and preserve critical structures such as the orbit or skull base [6-8].

We present a case of SNUC initially presenting with ocular symptoms, including exophthalmos and diplopia, highlighting the importance of recognizing atypical presentations and stressing the role of multimodal therapy in achieving disease control.

## Case presentation

A 72-year-old Caucasian man presented with complaints of excessive lacrimation, right-sided exophthalmos, and intermittent diplopia. He had no history of tobacco use or occupational exposure to hazardous chemicals. His medical history was significant for hypertension and hyperlipidemia. Family history included prostate cancer in his father and colon cancer in his mother.

The patient reported initial nasal congestion in January 2024 during a COVID-19 infection, without epistaxis, facial pain, or visual changes. In April 2024, he developed persistent excessive lacrimation and conjunctival irritation that was refractory to topical eye treatments. Over subsequent weeks, he noted progressive right-sided exophthalmos and intermittent mild diplopia occurring one to two times per week in all directions of gaze. Despite these symptoms, he retained the ability to read and drive without difficulty. Ocular review was notable for blurred vision, diplopia, excessive lacrimation, and exophthalmos. Neurologic review revealed intermittent headaches but no dizziness, gait disturbances, sensory deficits, or focal weakness. The remainder of the review of systems was unremarkable. Vital signs were within normal limits except for elevated blood pressure (158/68 mmHg). On examination, the patient was alert and oriented, with no acute distress. Head and neck examination revealed right-sided exophthalmos with orbital expansion. Pupils were equal, round, and reactive to light. Extraocular movements were intact but limited on the right. Cranial nerves II through XII were intact otherwise. No cervical, supraclavicular, or axillary lymphadenopathy was appreciated. The remainder of the physical examination was unremarkable. Given the patient’s presentation with unilateral exophthalmos and diplopia, the differential diagnosis included Graves ophthalmopathy, orbital lymphoma, sinonasal squamous cell carcinoma, ocular melanoma, and traumatic or inflammatory orbital disease.

**Figure 1:**
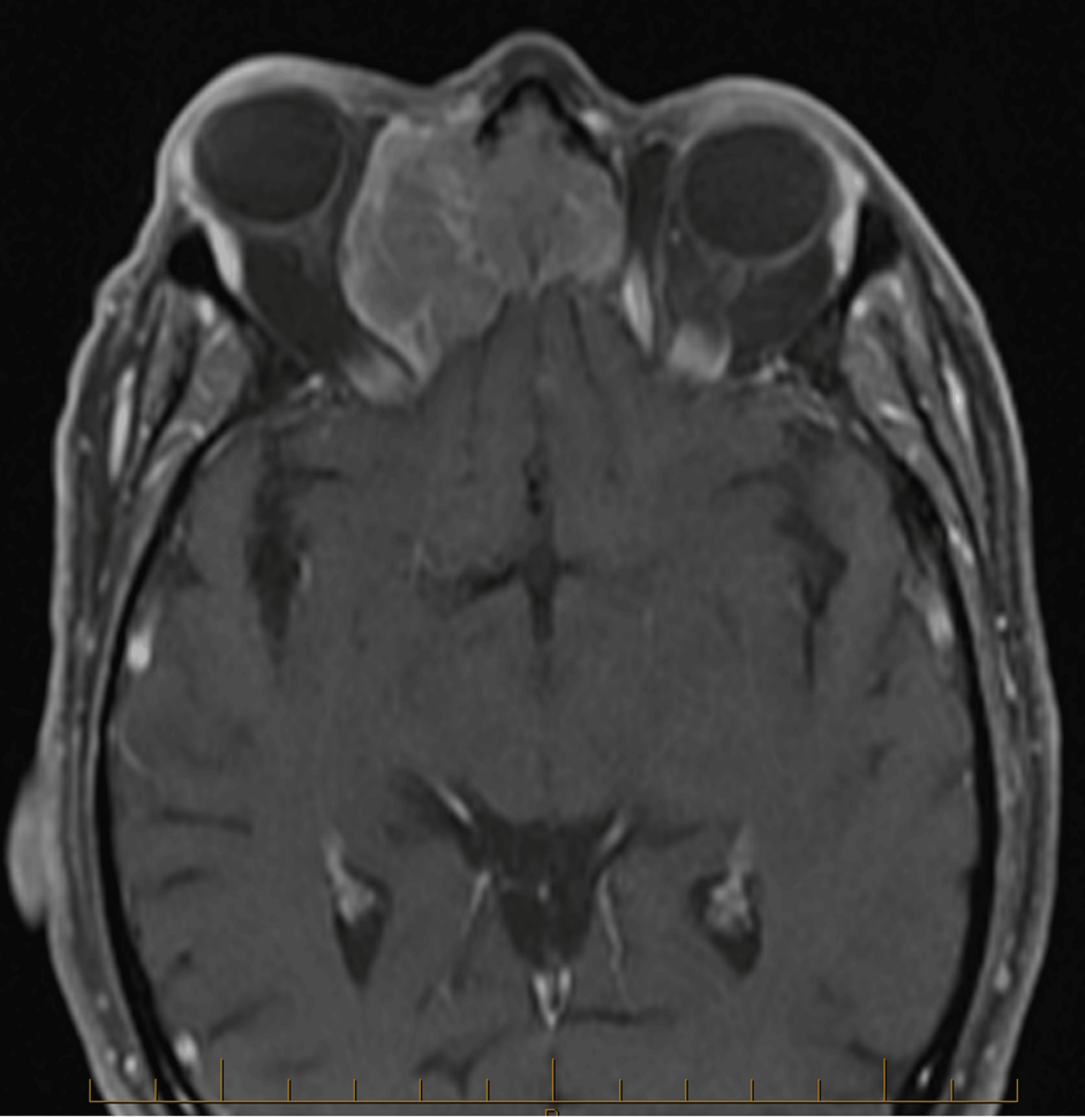
MRI orbit T1+c acquired on April 25, 2024. MRI: magnetic resonance imaging

Magnetic resonance imaging (MRI) of the face and sinuses performed on April 25, 2024, demonstrated a large right-sided sinonasal mass displacing the retro-orbital contents and globe, with extension into the ethmoid, maxillary, and frontal sinuses, as well as the nasal cavity (Figure 1).

**Figure 2:**
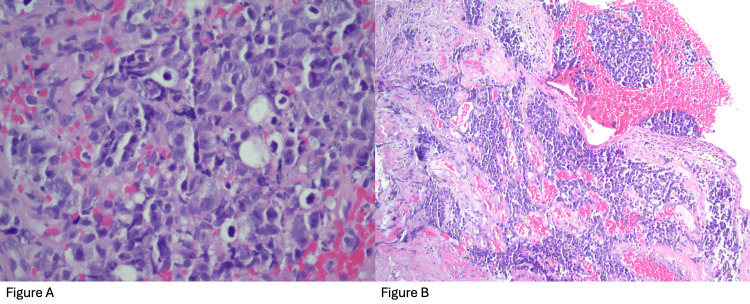
H&E-stained biopsy specimen showing poorly differentiated malignant cells consistent with sinonasal undifferentiated carcinoma. Hematoxylin and eosin (H&E)-stained sections of sinonasal undifferentiated carcinoma (SNUC). (A) High-power view demonstrating large malignant cells with prominent eosinophilic nucleoli, high nuclear-to-cytoplasmic ratio, and numerous mitotic figures. (B) Low-power view demonstrating a hypercellular small blue cell tumor with areas of tumor necrosis. Scale bars were unavailable on the original pathology images.

**Figure 3:**
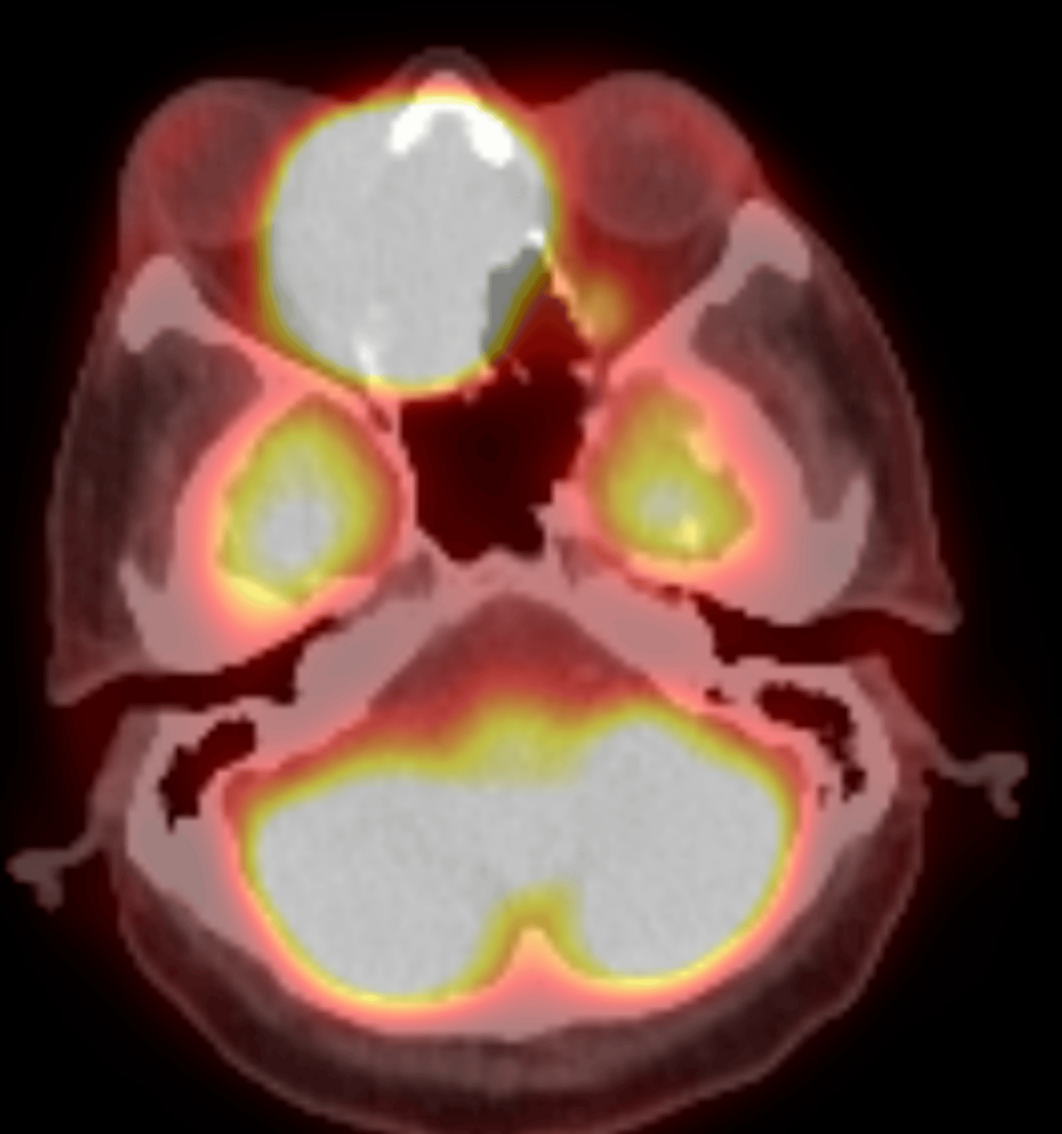
PET/CT imaging of sinonasal undifferentiated carcinoma (SNUC) acquired on May 5, 2024. PET/CT: positron emission tomography/computed tomography

An endoscopic biopsy was performed on April 26, 2024. Histopathologic examination showed sheets of atypical undifferentiated cells with high nuclear-to-cytoplasmic ratios and numerous mitotic figures (Figure 2). Immunohistochemical staining was positive for CK8/18 and pancytokeratin and negative for p16, CD45, chromogranin, and synaptophysin, supporting the diagnosis of SNUC. Representative immunohistochemical images and pathology scale bars were not available for retrospective inclusion in this report. A positron emission tomography/computed tomography (PET/CT) scan on May 5, 2024, demonstrated intense uptake in the primary tumor, with no evidence of regional nodal involvement or distant metastases (Figure 3). The final diagnosis was a clinically staged T4 primary tumor without regional lymph node involvement (cT4N0M0) SNUC of the ethmoid sinus, classified according to the American Joint Committee on Cancer 8th edition staging system for sinonasal malignancies.

**Figure 4:**
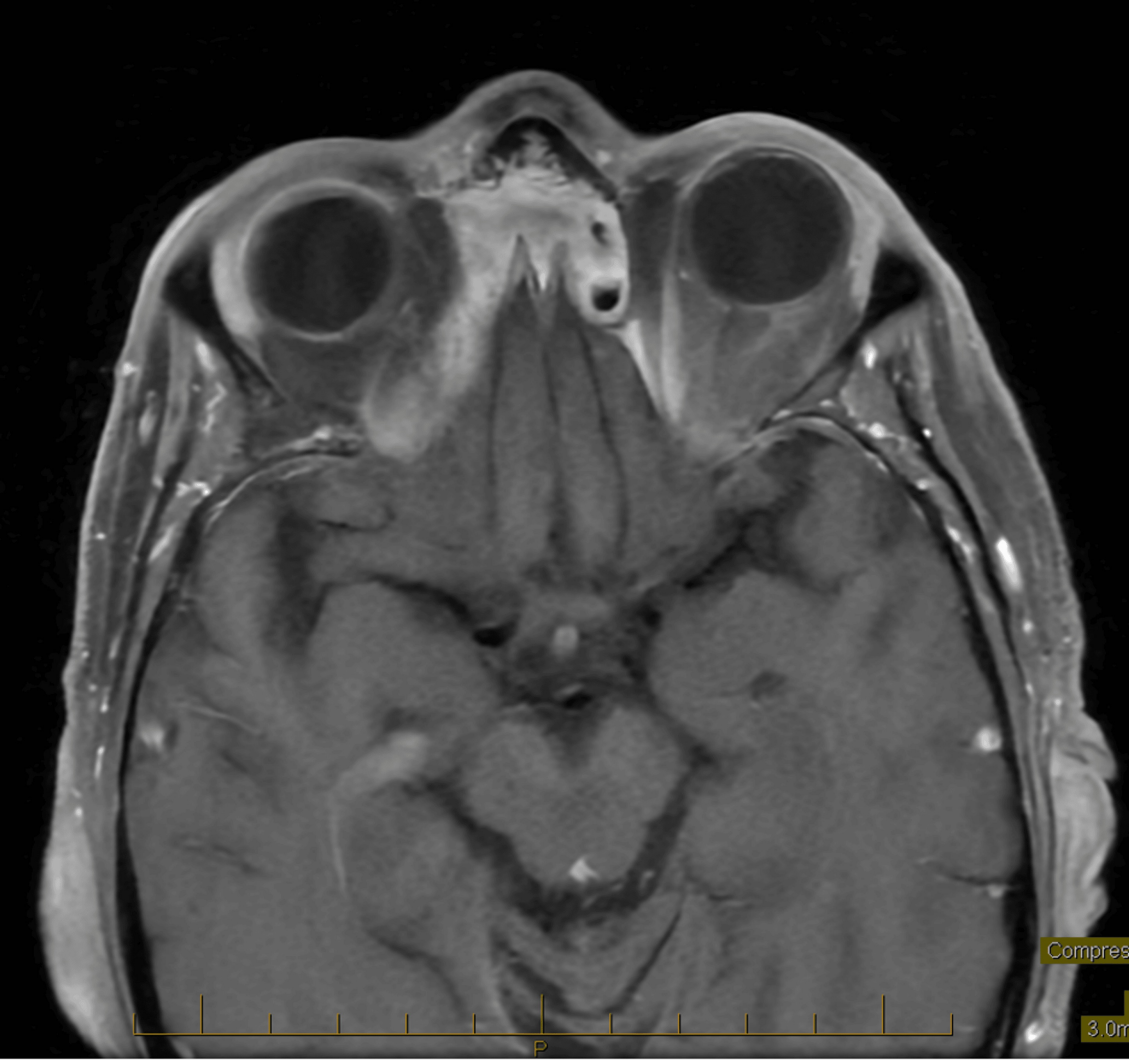
Post-chemotherapy MRI orbits T1+c (July 9, 2024). MRI: magnetic resonance imaging

The patient’s case was reviewed at a multidisciplinary tumor board, and a multimodal treatment approach was recommended (Table 1). Given the locally advanced disease with orbital involvement, induction chemotherapy with cisplatin and docetaxel was selected based on multidisciplinary consensus and institutional treatment practices, with the goals of reducing tumor burden, assessing chemosensitivity, and facilitating organ preservation while maintaining treatment tolerability. The patient received three cycles beginning May 13, 2024, consisting of cisplatin and docetaxel at 75 mg/m² every 21 days. Post-induction chemotherapy MRI on July 9, 2024, demonstrated a significant reduction in tumor size (Figure 4).

**Table 1:** Chronological timeline of clinical presentation, diagnosis, and treatment course. MRI: magnetic resonance imaging

Date	Clinical event	Findings/outcome
January 2024	Initial symptoms during COVID-19 infection	Developed nasal congestion without epistaxis, facial pain, or visual symptoms
April 2024	Progression of symptoms with ocular symptom development	Developed excessive lacrimation and conjunctival irritation refractory to topical treatment, leading to progressive right-sided exophthalmos and intermittent diplopia
April 25, 2024	Magnetic resonance imaging (MRI) of the face and sinuses	Revealed a large right-sided sinonasal mass extending into the ethmoid, maxillary, and frontal sinuses with orbital displacement
April 26, 2024	Endoscopic biopsy performed	Histopathology and immunohistochemistry confirmed sinonasal undifferentiated carcinoma (SNUC)
May 5, 2024	Positron emission tomography/computed tomography (PET/CT)	Demonstrated intense uptake in the primary tumor without nodal or distant metastatic disease
May 2024	Clinical staging	Diagnosed as locally advanced sinonasal undifferentiated carcinoma of the ethmoid sinus, clinically staged as cT4N0
May 13, 2024	Initiation of induction chemotherapy	Began cisplatin and docetaxel every 21 days for three cycles
July 9, 2024	Post-induction MRI	Demonstrated a significant reduction in tumor size
July 12, 2024	Radiation therapy simulation	Prepared for definitive concurrent chemoradiation
July 22-September 5, 2024	Concurrent chemoradiation therapy	Received intensity-modulated radiation therapy (IMRT) with weekly cisplatin
November 6, 2024	Post-treatment PET imaging	Demonstrated complete metabolic response
August 20, 2025	Follow-up MRI	Showed minimal enhancement without clear evidence of recurrence
December 3, 2025	PET/CT from most recent follow-up	Patient remained disease-free with manageable treatment-related toxicities

**Figure 5:**
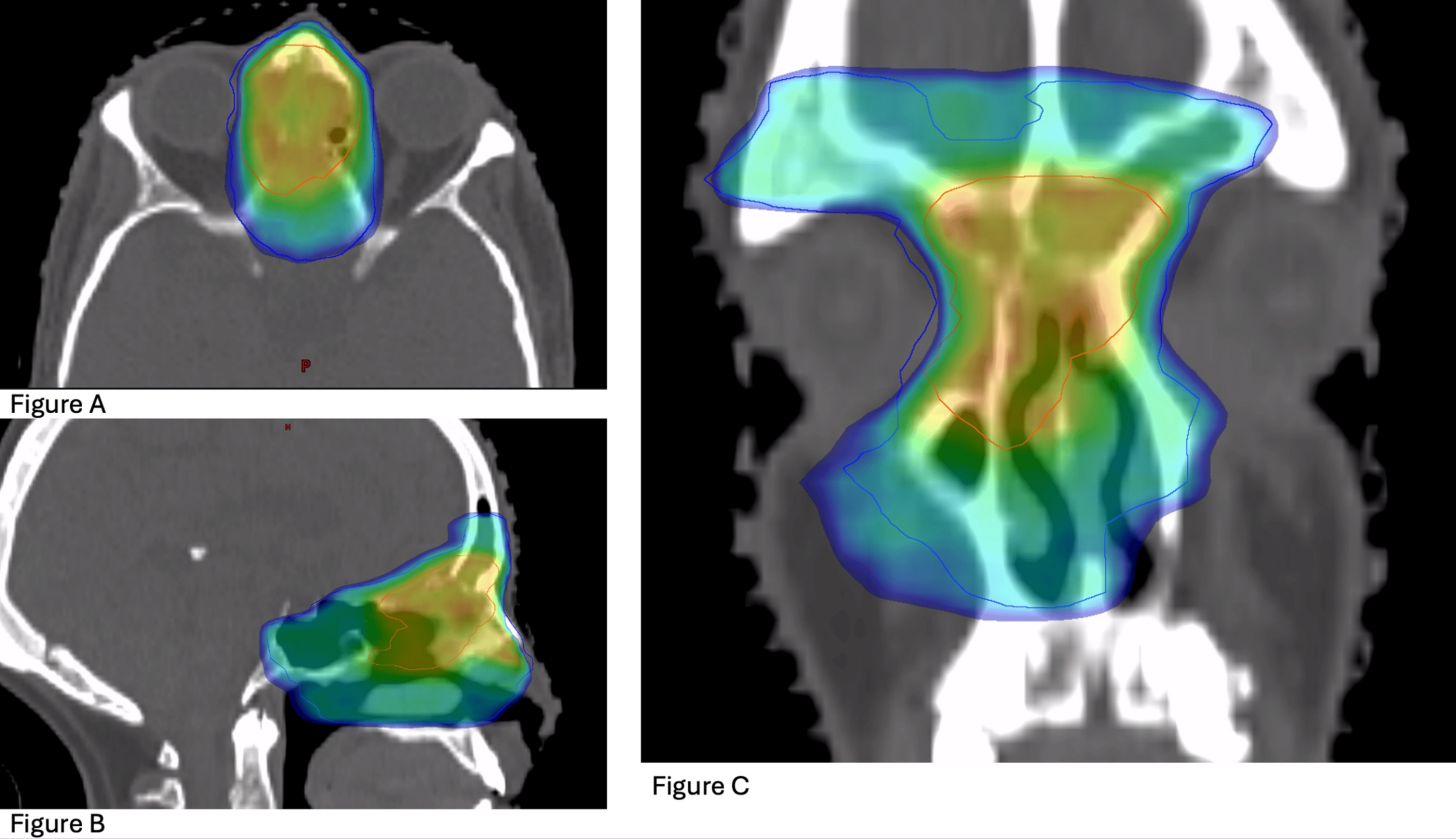
Radiotherapy target volume delineation for sinonasal undifferentiated carcinoma. Axial (A), coronal (B), and sagittal (C) contrast-enhanced CT images from radiation treatment planning demonstrate target volume contouring of the primary sinonasal mass. CT: computed tomography

Following induction therapy, the patient underwent radiation therapy simulation on July 12, 2024. From July 22, 2024, through September 5, 2024, he received definitive concurrent chemoradiation consisting of intensity-modulated radiation therapy (IMRT) delivering 66 Gy in 33 fractions (Figure 5) with weekly cisplatin at 40 mg/m² for six cycles. Target volumes were defined using pre-treatment MRI and PET/CT imaging findings. Radiation treatment planning incorporated standard organ-at-risk constraints when feasible, given the extent of local invasion and the goal of orbital preservation, with efforts to minimize radiation exposure to adjacent critical structures, including the optic apparatus and orbital contents. The prescribed dose of 66 Gy was selected in accordance with definitive treatment approaches for locally advanced sinonasal malignancies. Detailed retrospective dosimetric parameters, including specific optic pathway, lens, and chiasm dose constraints, as well as adaptive replanning data, were not available for inclusion in this report. He was monitored closely by ophthalmology and audiology for treatment-related toxicities.

**Figure 6:**
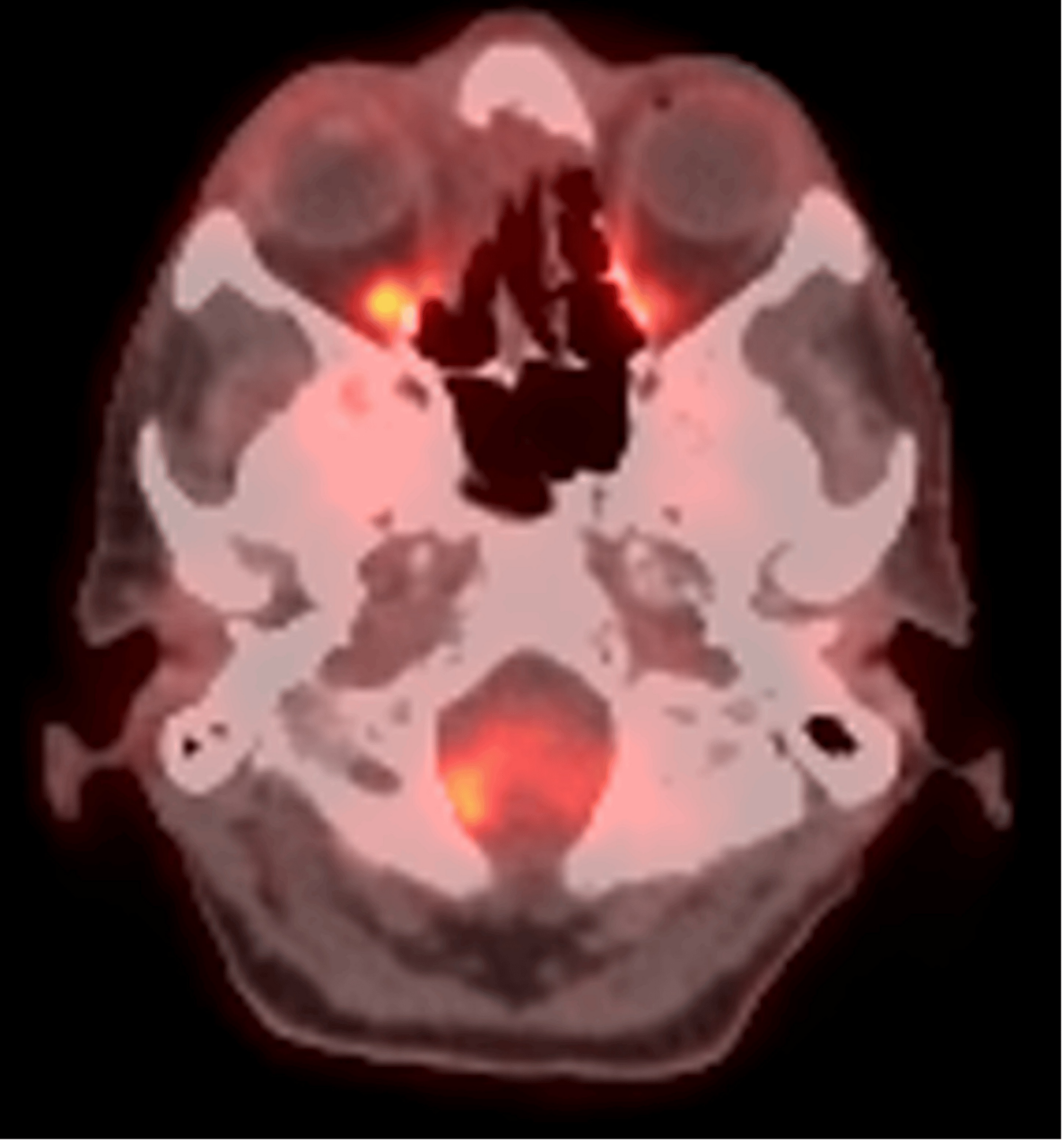
Post-treatment PET acquired on November 6, 2024. PET: positron emission tomography

**Figure 7:**
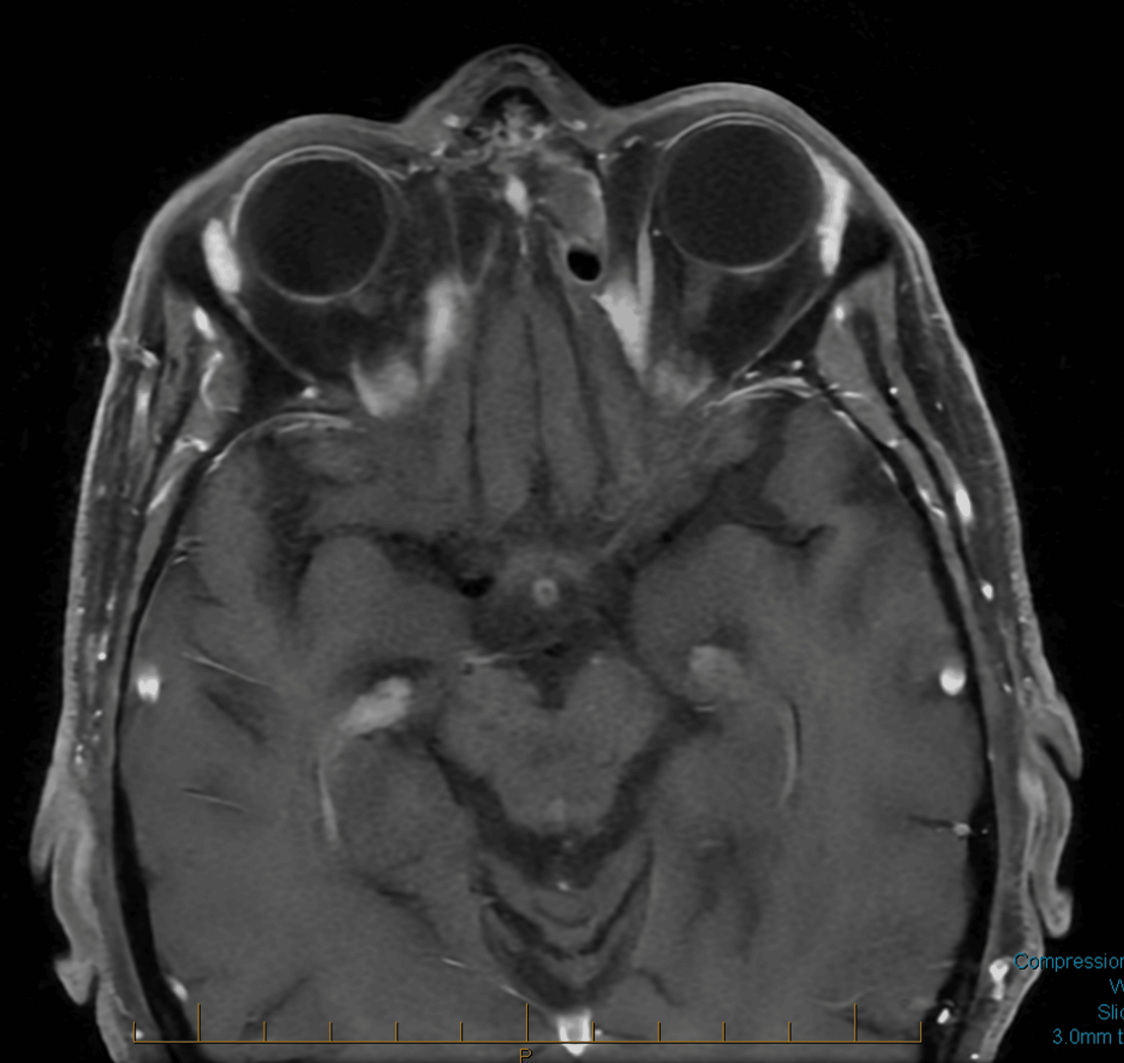
Follow-up MRI orbits (August 20, 2025). MRI: magnetic resonance imaging

Post-treatment PET imaging performed on November 6, 2024, demonstrated a complete metabolic response (Figure 6). Follow-up MRI in August 2025 showed minimal enhancement along the paramedian aspect of the left anterior ethmoid air cells without clear evidence of recurrence (Figure 7).

**Figure 8:**
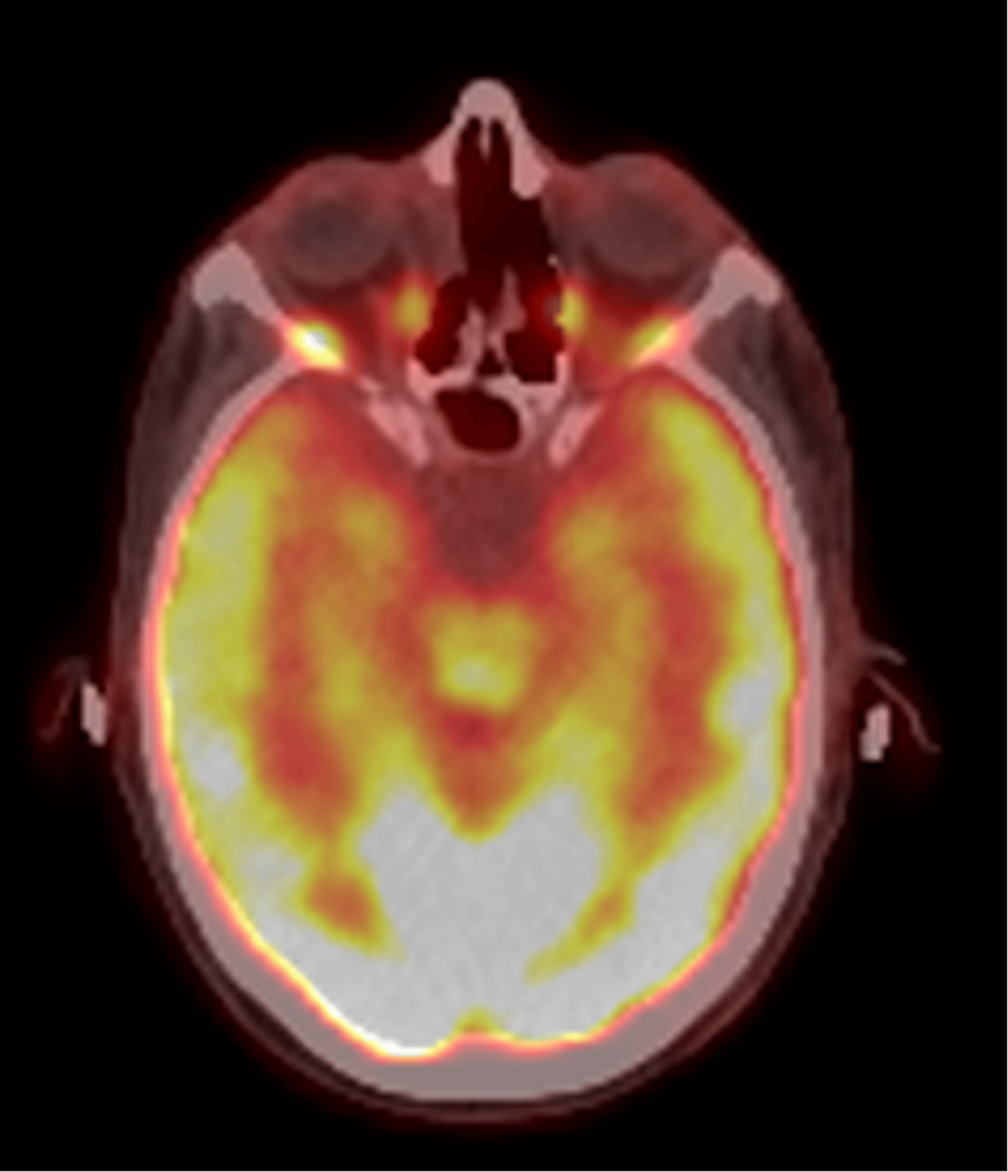
PET-CT from December 3, 2025. PET-CT: positron emission tomography-computed tomography

At his most recent follow-up in December 2025, the patient’s PET scan showed that he remained disease-free (Figure 8). He reported persistent but manageable radiation-related toxicities, including nasal congestion, eye dryness, complete anosmia, and mild hearing loss that improved with hearing aids. He underwent septoplasty and bilateral submucosal resection of the inferior turbinates with subsequent improvement in nasal airflow. 

## Discussion

SNUC is a rare, highly aggressive epithelial malignancy arising from the Schneiderian mucosa of the sinonasal tract, accounting for approximately 3-5% of sinonasal cancers, with an estimated incidence of 0.2 per 100,000 individuals. Patients frequently present with advanced local disease due to the nonspecific nature of early symptoms, contributing to historically poor outcomes [2-3].

Orbital involvement, including exophthalmos and diplopia, is a recognized but relatively uncommon presenting feature and often signifies locally advanced disease with extension beyond the sinonasal cavity. Previous series have demonstrated that invasion of adjacent critical structures, such as the orbit, skull base, or intracranial compartment, is associated with a worse prognosis and increased treatment-related morbidity [4]. In the present case, ocular symptoms served as the initial clinical clue prompting advanced imaging and diagnostic evaluation.

Histopathologically, SNUC is characterized by sheets of poorly differentiated malignant epithelial cells with high mitotic activity, necrosis, and frequent vascular or neural invasion. Accurate diagnosis is difficult due to considerable overlap with other sinonasal malignancies, including olfactory neuroblastoma, lymphoepithelial carcinoma, and nasopharyngeal-type undifferentiated carcinoma. Therefore, comprehensive immunohistochemical evaluation is essential, as Jeng et al. demonstrated that SNUC is a clinically and biologically distinct entity requiring different therapeutic considerations [5].

Recent advances in molecular characterization have further refined the understanding of SNUC biology. Genetic analyses have identified recurrent *IDH2* mutations, alterations in the SWI/SNF complex, such as SMARCB1 loss, and rare gene fusions, including PGAP3-SRPK1, suggesting biologically distinct subtypes bearing potential implications for future targeted therapies [9]. Although molecular testing was not performed in this patient, these molecular insights are not yet routinely incorporated into treatment algorithms but continue to highlight the evolving landscape of SNUC management and may influence future therapeutic strategies.

Given the aggressive nature of SNUC and the absence of standardized treatment guidelines, multimodal therapy continues to be the cornerstone of management. Historical approaches emphasizing surgery alone or radiotherapy alone have yielded poor survival outcomes. Contemporary evidence supports combined modality treatment incorporating chemotherapy and radiation, with or without surgery, to improve local control and survival [6]. However, management strategies remain heterogeneous, particularly in patients with orbital or skull base involvement, where surgical morbidity may be substantial.

More recently, induction (neoadjuvant) chemotherapy has emerged as a promising strategy for patients with locally advanced SNUC. Amit et al. [7] demonstrated that response to induction chemotherapy is predictive of outcomes and can guide subsequent treatment decisions, including the feasibility of organ-preserving approaches. A comprehensive review by Melder and Geltzeiler [8] further supports induction chemotherapy as a key component of modern SNUC management, particularly in cases with orbital or skull base involvement, where surgical morbidity is high.

In this case, the patient’s disease was reviewed at a multidisciplinary tumor board, and treatment decisions were individualized based on the extent of locally advanced disease with orbital involvement. A nonsurgical organ-preserving approach was favored to minimize operative morbidity while preserving orbital function. Induction chemotherapy with cisplatin and docetaxel was selected based on multidisciplinary consensus and institutional treatment practices, with the goals of reducing tumor burden, assessing chemosensitivity, and facilitating definitive concurrent chemoradiation. Although other induction regimens, including TPF (a chemotherapy combination regimen consisting of T = docetaxel (Taxotere), P = cisplatin (platinum agent), F = fluorouracil (5-FU))-based and platinum-etoposide combinations, have been described in the literature, there remains no universally accepted standard regimen for SNUC.

The patient described in this case was treated with induction chemotherapy followed by definitive concurrent chemoradiation, achieving a complete radiographic and metabolic response. This outcome is consistent with emerging evidence suggesting that induction chemotherapy may facilitate tumor debulking, improve radiation targeting, and support organ-preserving treatment strategies in select patients with locally advanced disease [7-8]. Treatment-related toxicities were monitored clinically with ophthalmologic and audiologic follow-up throughout therapy. Despite ongoing treatment-related sequelae, the patient remains disease-free at long-term follow-up, highlighting the potential benefit of individualized multimodal management approaches in SNUC.

## Conclusions

SNUC remains a rare and highly aggressive malignancy with historically poor outcomes, largely due to advanced stage at diagnosis and frequent invasion of adjacent critical structures. While multimodal therapy is widely accepted as the most effective treatment strategy, the optimal sequencing of surgery, chemotherapy, and radiation remains undefined. Increasing evidence supports the use of induction chemotherapy in locally advanced disease, both as a prognostic tool and as a means of facilitating organ preservation. Recent National Comprehensive Cancer Network Head and Neck Cancer Guidelines support consideration of multimodal treatment approaches, including induction chemotherapy in select patients with locally advanced sinonasal malignancies. Advances in molecular profiling, including the identification of *IDH2 *mutations and alterations in the SWI/SNF complex, offer promising avenues for future targeted therapies but have yet to be integrated into routine clinical practice. Early recognition of atypical orbital manifestations and prompt multidisciplinary evaluation remain important for the timely diagnosis and treatment of SNUC. This case demonstrates a favorable response to induction chemotherapy followed by definitive concurrent chemoradiation with successful organ preservation in a patient with locally advanced disease. These findings are consistent with emerging literature supporting individualized multimodal treatment approaches for select patients with SNUC.
